# Involvement of Src family of kinases and cAMP phosphodiesterase in the luteinizing hormone/chorionic gonadotropin receptor-mediated signaling in the corpus luteum of monkey

**DOI:** 10.1186/1477-7827-10-25

**Published:** 2012-03-29

**Authors:** Shah B Kunal, Asaithambi Killivalavan, Rudraiah Medhamurthy

**Affiliations:** 1 Department of Molecular Reproduction, Development and Genetics, Indian Institute of Science, Bangalore 560012, India

**Keywords:** cAMP-Phosphodiesterase (PDE), Corpus luteum, (LH/CGR), SR-B1, Src family of kinases (SFKs)

## Abstract

**Background:**

In higher primates, during non-pregnant cycles, it is indisputable that circulating LH is essential for maintenance of corpus luteum (CL) function. On the other hand, during pregnancy, CL function gets rescued by the LH analogue, chorionic gonadotropin (CG). The molecular mechanisms involved in the control of luteal function during spontaneous luteolysis and rescue processes are not completely understood. Emerging evidence suggests that LH/CGR activation triggers proliferation and transformation of target cells by various signaling molecules as evident from studies demonstrating participation of Src family of tyrosine kinases (SFKs) and MAP kinases in hCG-mediated actions in Leydig cells. Since circulating LH concentration does not vary during luteal regression, it was hypothesized that decreased responsiveness of luteal cells to LH might occur due to changes in LH/CGR expression dynamics, modulation of SFKs or interference with steroid biosynthesis.

**Methods:**

Since, maintenance of structure and function of CL is dependent on the presence of functional LH/CGR its expression dynamics as well as mRNA and protein expressions of SFKs were determined throughout the luteal phase. Employing well characterized luteolysis and CL rescue animal models, activities of SFKs, cAMP phosphodiesterase (cAMP-PDE) and expression of SR-B1 (a membrane receptor associated with trafficking of cholesterol ester) were examined. Also, studies were carried out to investigate the mechanisms responsible for decline in progesterone biosynthesis in CL during the latter part of the non-pregnant cycle.

**Results and discussion:**

The decreased responsiveness of CL to LH during late luteal phase could not be accounted for by changes in LH/CGR mRNA levels, its transcript variants or protein. Results obtained employing model systems depicting different functional states of CL revealed increased activity of SFKs [pSrc (Y-416)] and PDE as well as decreased expression of SR-B1correlating with initiation of spontaneous luteolysis. However, CG, by virtue of its heroic efforts, perhaps by inhibition of SFKs and PDE activation, prevents CL from undergoing regression during pregnancy.

**Conclusions:**

The results indicated participation of activated Src and increased activity of cAMP-PDE in the control of luteal function *in vivo*. That the exogenous hCG treatment caused decreased activation of Src and cAMP-PDE activity with increased circulating progesterone might explain the transient CL rescue that occurs during early pregnancy.

## Background

The primary function of corpus luteum (CL) is to secrete progesterone (P_4_), essential for establishment and/or maintenance of pregnancy in mammals [1,2]. The structure and function of CL are controlled by luteotrophic factors (stimulate growth and function) and luteolytic factors (cause functional and structural regression). It is increasingly becoming apparent that there exists a large diversity in the regulation of CL function not only among species, but also within species at different stages of the luteal phase dictated largely by the intricate interplay between luteotrophic and luteolytic factors. In higher primates, one of the important characteristics of the regulation of CL function is that unlike other species, circulating LH appears to be the sole trophic factor responsible for its control during the non-fertile cycle [3]. Intriguingly, circulating P_4 _levels decline and the CL eventually undergoes regression at the end of non-fertile cycle despite the accompanying lack of decrease in LH levels [4,5]. On the other hand, during pregnancy the increasing production of chorionic gonadotropin (CG) elaborated by the placenta arrests the decline in P_4 _secretion resulting in rescue of CL function [6]. Although recent studies continue to expand our current understanding of the cellular and molecular actions of LH/CG, knowledge of the processes whereby LH/CG promote development and maintenance of CL function is far from clear. Though LH and CG bind to a common LH/CG receptor (R), they activate different signal transduction pathways for maintenance of CL function as reported in spheroidal cell culture system of human granulosa lutein cells [7,8]. The LH/CGR is a G protein-coupled receptor (GPCR) involving activation of Gs, Gi/o and Gq/11 family of G proteins [9-12], but it is generally accepted that modulation of Leydig/luteal cell function is mediated primarily by the activation of the canonical Gs/cAMP/PKA/CREB pathway [13,14]. Analysis of cAMP/PKA/CREB and MAP kinase pathways in the luteal tissue has suggested fine tuning of luteal function during luteolysis and rescue of CL function, however, involvement of these pathways alone does not appear to fully account for the regulation of CL function [15]. Recently, several studies have described the phenomenon of activated GPCRs to crosstalk with or transactivate tyrosine kinase receptor signaling [16,17], and extensive studies in mouse Leydig tumor cells have demonstrated involvement of Src family of kinases (SFKs) in modulation of LH/CG responsiveness, and thereby regulation of Leydig cell function [18-21]. Eventhough the precise mechanism by which LH/CGR signaling causes activation of these kinases and the functional consequences of their activation remains to be determined, SFKs might have an important role in the regulation of CL function, perhaps by modulation of LH/CG responsiveness, particularly during the late luteal phase of non-fertile cycles. Thus, studies are essential to identify other signaling pathways to further delineate intraluteal processes to discern those that are critical to the control of luteal function and its life span.

To maintain high circulating P_4 _levels, steroid biosynthesis in luteal cells requires a constant supply of cholesterol [22]. The intracellular cholesterol trafficking from outer to inner mitochondrial membrane, regulated by StAR protein, has been extensively investigated [23,24]. On the other hand, the importance of cholesterol acquisition into luteal cell needs to be explored to further understand the process of substrate mobilization to the CL. It has been suggested that cholesterol is preferentially sourced from circulating high- and low- density lipoproteins (HDL and LDL) [25,26]. Unlike the classical LDL receptor pathway in which the entire lipoprotein is internalized, the trafficking of cholesterol from HDL involves binding of HDL-cholesteryl ester to scavenger receptor class B type 1 (SR-B1) located on the luteal cell, and only cholesteryl ester is delivered without the concomitant uptake and degradation of the entire HDL particle, leaving the lipoprotein at the cell surface [27-31]. The expression changes of SR-B1 and its transcriptional regulation by factors such as Liver receptor homologue 1 (LRH-1/NR5A2) and Steroidogenic factor 1 (SF-1/NR5A1) become crucial to understanding the regulation of CL function [32,33].

To further expand the knowledge on the regulation of CL function during spontaneous luteolysis in monkeys, studies have been carried out with following objectives: to (i) establish correlation between circulating P_4 _and the LH/CGR expression dynamics throughout the luteal phase (ii) determine the expression and activation of Src family of non-receptor tyrosine kinases and cAMP-phosphodiesterase (PDE), and (iii) to examine expression patterns of SR-B1 and genes associated with steroidogenesis in the CL.

## Methods

### Reagents

Trizol reagent and GenElute™ Gel Extraction Kit were purchased from Sigma-Aldrich Co., (St Louis, MO, USA). Oligonucleotide primers were synthesized by Sigma-Genosys (Bangalore, India). Gonadotropin releasing hormone receptor (GnRH/R) antagonist, Cetrorelix^® ^(CET) was a kind gift from Asta Medica (Frankfurt, Germany). Recombinant human LH (rhLH) and hCG (Profasi^®^) were from Ares-Serono (Aubonne, Switzerland). Labelled α-^32^P dCTP (LCP103) was from BRIT (Hyd., India) and Random Primer extension Labeling Kit (#KT04) was from Bangalore Genei (Bangalore, India). Polyclonal antibodies specific to LH/CGR (H-50: sc-25828), PDE4D (H-69: sc-25814) and ERK-2 (# sc-154) were from Santa Cruz Biotechnology Inc. (Santa Cruz city, CA, USA). Phospho-Src family (Y-416; #2101), Src Antibody sampler kit (#9935), p42/44 MAPK (# 9102), Horse radish peroxidase labeled goat anti-rabbit IgG and ECL chemiluminescence kit were from Cell Signaling Technology (Beverly, MA, USA). Polyclonal antibody against SR-B1 was a kind gift from Professor Salman Azhar (Stanford University School of Medicine). Snake venom nucleotidase (*Crotalus atrox *venom) was procured from Enzo Life Sciences, UK. Nylon and PVDF membranes were purchased from NEN life Sciences, (Boston, MA, USA). All other reagents were from Sigma-Aldrich Co., (Bangalore, India) or sourced from local distributors.

### Animal protocols, blood samples, and CL collection

Experimental protocols in the monkeys described here were approved by the Institutional Animal Ethics Committee of the Indian Institute of Science. The general care and housing of monkeys at the Primate Research Laboratory, Indian Institute of Science, Bangalore, have been described elsewhere [13]. Adult female bonnet monkeys (*Macaca radiata*) weighing 3.3 to 5.1 kg with a history of regular menstrual cyclicity (27-29 days) were utilized for the study. Monkeys were monitored daily for the onset of menses, and blood samples through femoral venipuncture were collected daily from day 8-12 of the menstrual cycle for determining the day of onset of E_2 _surge. Further, blood samples were collected either daily or at more frequent intervals until the time of CL retrieval or onset of menses. In this study, one day after the occurrence of E_2 _peak was designated as day 1 of the luteal phase. The CL tissues on the designated day of the luteal phase or at the end of treatment protocol (see below) were retrieved from the ketamine hydrochloride 15 mg/kg BW and/or pentobarbital sodium (8 mg/kg BW) anesthetized monkeys subjected to laparotomy under aseptic conditions. The CL retrieved was transferred to a sterile petri dish containing filter paper, cut into four or more pieces, a single piece each processed for cAMP and PDE assays. The remaining pieces were placed in individual sterile cryovials and snap frozen in liquid nitrogen before storing at -70°C.

### Experiment 1: Expression of LH/CGR, genes belonging to SFKs, cAMP-PDE and SR-B1 in the monkey CL throughout the luteal phase

CL tissues (n = 3/stage) were collected from monkeys on day 5 [early (E)], day 8 [mid (M)], and on day 14 [late (L)] of the luteal phase as described previously [15,34]. In addition, CL tissues (n = 3) were collected from monkeys on day 1 of menses (D1M), a time point when luteolytic events are considered to be manifest. Blood samples were collected on designated days, to determine the steroidogenic status of CL. The tissues retrieved during different developmental stages were used to examine the expression of genes considered important for the modulation of the molecular events that regulate CL structure and function during the luteal phase.

### Experiment 2: Effect of PGF_2α _treatment on expression of LH/CGR, genes belonging to SFKs and SR-B1 in the monkey CL

PGF_2α _treatment induces luteolysis in various species without affecting the circulating LH levels [35]. An experimental regimen has been standardized in the laboratory in which three injections of Iliren, a synthetic analogue of PGF_2α _(59 μg/kgBW) were administered at 8 h intervals on day 10 of the luteal phase and CL tissues (n = 3) were collected 24 h after initiation of treatment [34]. Similarly, on a separate occasion, the same monkeys received three injections of PBS at 8 h intervals beginning on day 10 of the luteal phase and CL tissues were collected 24 h later to serve as control (VEH). Blood samples were collected at different intervals before and after treatment. The effect of PGF_2α _on expression of various genes was examined.

### Experiment 3: Expression and regulation of LH/CGR, genes belonging to SFKs and SR-B1 in the monkey CL during induced luteolysis (LH secretion inhibition) and rescue of CL function (LH replacement)

To induce luteolysis, monkeys were administered with GnRH-R antagonist, CET at a dose of 150 μg/kgBW s.c., on day 7 of the luteal phase, similarly on separate occasions, the same monkeys received vehicle (5.25% glucose; VEH) and CL tissues were collected 24 h after CET/VEH treatment. Further, on day 8 of the luteal phase, six monkeys treated with CET for 24 h were further treated with either PBS (VEH, n = 3 monkeys) or rhLH 20 IU/kgBW (rhLH, n = 3) i.v., and CL tissues were collected at the end of 1 and 8 h rhLH treatment [34]. Blood samples were collected at various time points before and after treatment/s essentially as per the earlier reported protocol [34]. The tissues obtained after LH secretion inhibition and rescue of CL function by LH replacement were subjected to examination of various genes, SFKs activation and cAMP levels.

### Experiment 4: Expression and regulation of LH/CGR, genes belonging to SFKs and SR-B1 in CL during simulated early pregnancy

It is established that events associated with rescue of CL function that occur during early pregnancy could be simulated in non-fertile monkeys by administration of incremental doses of hCG [5]. To examine the expression of LH/CGR, genes belonging to SFKs and SR-B1 during simulated early pregnancy, monkeys were administered hCG in incremental doses from day 9-13 of the luteal phase (15, 30, 45, 90, and 180 IU, twice daily, i.m.) and CL tissues were retrieved on d 14 of the luteal phase as per the protocol previously reported [5]. For comparison, CL collected on day 14 (late CL-L) of the luteal phase but without hCG treatment, was used as the control.

### Hormone assays

Serum P_4 _concentrations were determined by specific RIAs as reported previously [36]. The sensitivity of P_4 _assay was 0.1 ng/ml, and the inter- and intra-assay variations were < 10%.

### Preparation of CL tissue samples for cAMP assay

Fresh CL tissues collected from different experiments were boiled immediately in boiling water (95°C) bath for 10 min to inactivate the PDE enzyme and then homogenized in sterile H_2_O and centrifuged at 30,000 × g for 15 min. The supernatants were assayed for cAMP and to increase the sensitivity of the assay, samples were acetylated i.e., 100 μl of supernatant was made up to 1.5 ml final volume using 1X PBS, 30 μl of triethylamine and 15 μl of acetic anhydride and mixed properly for acetylation process to complete. These acetylated samples were used for cAMP RIA.

### Radioimmunoassay for cAMP

The assay procedure was standardized in the laboratory as per the protocol of National Hormone & Peptide Program (NHPP) and Steiner et.al., 1969 [37] The sensitivity for cAMP assay was ~0.01 pmoles/tube and the inter- and intra-assay variations were 5.2% and 7.4% respectively. The antibody (CV-27) was used at the final assay tube dilution of 1: 200,000 in a total assay set up volume of 500 μl. The standard graph constructed with concentrations of different acetylated standard on X-axis and % binding on Y-axis was used to extrapolate concentration of cAMP in samples.

### Determination of PDE activity using^3^HcAMP as substrate

The cAMP-PDE activity assay was carried out as reported previously with few modifications [38]. Fresh CL tissues (8-10 mg) were homogenized in 200 μl of PDE lysis buffer and the lysate containing enzyme was incubated with cocktail containing^3^HcAMP substrate, 50 mM HEPES (pH 7.4) and 10 mM MgCl_2 _at 30°C for 15 min. The PDE reaction was stopped by incubating the tubes in 95°C hot water bath for 3 min and incubated again at 30°C for 15 min. To the reaction mixture, 10 μl (50 KU) of snake venom nucleotidase was added and incubated at 30°C for further 45 min. The reaction mixture was applied on to 1 ml syringe columns containing anion-exchange resin QAE sephadex A-25 (200-400 mesh, GE life sciences, NJ, USA) and equilibrated in 20 mM ammonium formate at pH 6.0 prior to use. The elution was carried out with 500 μl of 10 mM Tris pH 7.4, and the same was repeated for further 5 more times. The eluant was mixed with 10 ml of scintillation fluid and the dpm in each sample was counted for 5 min in liquid scintillation counter.

### RNA isolation

Total RNA was isolated from CL tissues harvested from different experiments using TRI Reagent according to manufacturer's instructions. RNA quantitation was performed using NanoDrop ND-1000 UV-VIS spectrophotometer (NanoDrop Products, Wilmington, DE) and RNA samples with A_260_/A_280 _values of ~1.8-1.9 were considered for further analysis.

### Semi-quantitative RT-PCR analysis

Semi-quantitative RT-PCR analysis was carried out essentially as described previously [39]. The details of primers employed along with the annealing temperature and expected amplicon size are provided in Additional file 1: Table S1.

### Real-time quantitative RT-PCR analysis

Real-time quantitative RT-PCR (qPCR) analysis was carried out essentially as described previously [34]. Expression of L19 was used as internal control for normalization of each gene examined. The list of genes with details of the primers employed along with the annealing temperature and expected amplicon size are provided in Additional file 2: Table S2.

### Northern blot analysis

Northern blot analysis was carried out as described previously with few modifications [40]. Briefly, total RNA (15 μg) extracted from CL were electrophoresed and blotted onto nylon membrane by capillary blotting for 16 h in 20× SSC. The blot was pre-hybridized for 2 h and hybridized with α^32^P-dCTP labeled LH/CGR cDNA probe for 18 h at 65°C in Church buffer [41]. The blots were then washed sequentially with buffers containing varying concentrations of SSC + SDS and then processed for autoradiography with an intensifying screen. Hybridization signals were quantitated using a PhosphorImager (Typhoon 9210, Amersham biosciences). The equal loading of RNA samples was confirmed by reprobing the stripped blot with L-19 labelled cDNA probe.

### Immunoblot analysis

CL tissue lysate preparation and immunoblot analysis were carried out following the previously published procedures with some modifications [40]. Briefly, nonspecific sites on the membrane were blocked using 10% milk in TBST by incubating 1 h at room temperature instead of overnight at 4°C. Membrane was further incubated with primary antibody specific for different proteins for overnight at 4°C instead for 3 h at room temperature. The membranes were developed by ECL chemiluminescence kit from Cell Signaling Technology (Beverly, MA) instead of (NEN Life Sciences). Autoradiographs were scanned using HP scanner or Syngene gel documentation system and quantitated by densitometry using Gene tools software (Syngene laboratories, MD, USA) instead of UVI-Tech gel documentation system and quantitated using UVI-Band Map (1999) software.

### Statistical analyses

Data were expressed as mean ± SEM. Statistical evaluation of mean differences in P_4 _concentrations and qPCR expression of genes was analysed by one-way ANOVA, followed by the Newman- Keuls multiple comparison test (PRISM GraphPad, version 5.0; GraphPad Software Inc., San Diego, CA) and Student's t-test to compare between two groups. A p value of < 0.05 was considered statistically significant.

## Results

### Expression and characterization of LH/CGR transcript variants and protein in the monkey CL throughout the luteal phase

**Figure 1:**
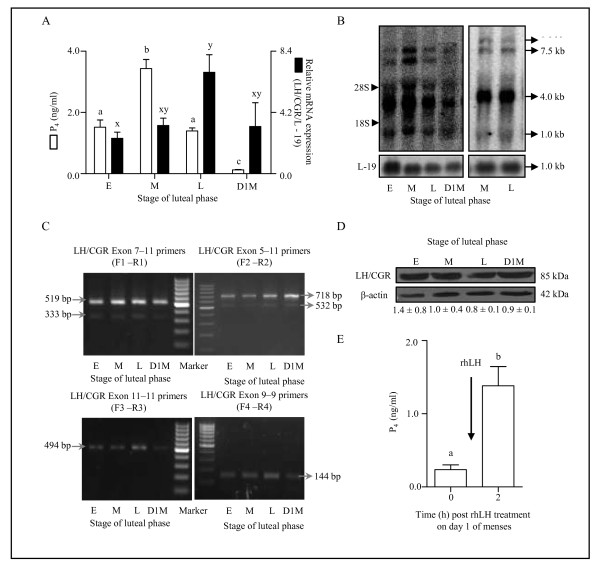
**Characterization of LH/CGR transcript variants and protein in the monkey CL throughout the luteal phase**. (A) Circulating mean serum P_4 _levels and LH/CGR mRNA expression determined during different stages of luteal phase. Individual bars represent mean ± SEM values (n = 3 animals/stage). For comparison among different stages of luteal phase, one-way ANOVA analysis is performed and bars with different letters are significantly (p < 0.05) different. (B) Northern blot analysis of LH/CGR mRNA in CL obtained from different stages of the luteal phase and during regression. Four transcripts of LH/CGR mRNA migrating with apparent sizes of 1.0, 4.0, 7.5 and 8.0 kb are indicated by arrows. The 28S and 18S ribosomal RNA corresponding to the sizes of 5 kb and 1.86 kb, respectively are also shown. A comparative analysis of LH/CGR mRNA expression between mid and late stage of luteal phase indicated presence of all four transcripts in the late stage CL. The blots were reprobed with α ^32^P labeled L-19 cDNA probe to confirm for equal loading of total RNA. The data shown is for one of three northern blots (n = 3 CL/stage). (C) Semi-quantitative RT-PCR analysis of the LH/CGR mRNA in CL tissues collected during different stages of the luteal phase. In the upper panel, representative gel pictures of the products obtained after employing two sets of primer pairs designed spanning exon 5-11 and exon 7-11 regions, respectively. In the lower panel, representative gel pictures of the products obtained employing two sets of primer pairs designed spanning exon 11 and exon 9 regions, respectively. The positions of amplified products are indicated by arrows. (D) Levels of LH/CGR protein in the monkey CL throughout the luteal phase. The immunoblot probed with anti-β-actin antibody was utilized to confirm that the equal amount of protein was loaded in each lane. Densitometric analysis (mean ± SEM) of immunoblots was performed and the protein level in the mid luteal phase was set as one fold and protein levels in other stages relative to the mid luteal phase are indicated below the respective bands. (E) Circulating P_4 _levels before and 2 h after rhLH (20 IU/kg BW) treatment on day 1 of menses. Each bar represents mean ± SEM values of six independent experiments. Different letters on bars indicate statistical significance (p < 0.05).

Circulating mean serum P_4 _levels increased from early to mid stage luteal phase (1.5 ± 0.2 vs. 3.4 ± 0.3 ng/ml), however, P_4 _levels were low at the late luteal phase (1.4 ± 0.1 ng/ml) and were 0.12 ± 0.01 ng/ml on day 1 of menses (Figure 1A). The qPCR analysis of LH/CGR expression at different stages of luteal phase revealed that the mRNA expression increased progressively to reach maximum at the late stage CL (2.4 ± 0.4, 3.3 ± 0.5 and 6.9 ± 1.2 ng/ml at early, mid and late stage, respectively Figure 1A). Surprisingly, detectable expression of LH/CGR mRNA was observed in CL collected on day 1 of menses (Figure 1A).

Northern blot analysis was performed for LH/CGR mRNA to examine the presence of different transcripts and their size in the monkey CL throughout the luteal phase. As seen in Figure 1B, the alternative splicing for LH/CGR mRNA was operational in the monkey CL and four transcripts were detected, one major band with an apparent size of 4.0 kb and three minor bands with sizes of 1.0, 7.5 and 8.0 kb. All the four transcripts were present in the CL tissues collected from monkeys at the late luteal phase and on day 1 of menses, but the intensity of transcripts was lower compared to mid stage (Figure 1B). Since LH/CGR mRNA expression was maximal at late luteal phase, it became of interest to examine whether any of these transcript variants were differentially regulated. The results indicated presence of similar expression of splice variants both during mid and late stage of the luteal phase (Figure 1B).

In contrast to the earlier report suggesting the absence of LH/CGR mRNA expression in the regressing monkey CL [42], in the present study the presence of LH/CGR mRNA expression in the regressing CL prompted us to employ multiple primer pairs PCR approach to further determine the nature of alternative splicing variants. The organization of LH/CGR gene and design of multiple primer pair sets and various predicted transcript variants lacking different exons are provided in Additional file 3: Figure S1. The sets of multiple primer pairs were designed such that both full length transcripts and splice variants would be detected. The details of primer sets, RT-PCR conditions and the expected size of splice variants are described in the Additional file 1: Table S1. Semi-quantitative RT-PCR analysis of LH/CGR mRNA demonstrates presence of two products of sizes 519 bp and 333 bp using exon 7-11(F1-R1) primer set whereas, products of sizes 718 bp and 532 bp using exon 5-11(F2-R2) primer set corresponding to a full length transcript and one splice variant lacking some exon region using both the primer sets (Figure 1C). The four cDNA products [upper (519/718 bp) and lower (333/532 bp) band using both the primer sets] were gel eluted and sequenced. The nucleotide sequences were compared with Gen Bank database using sequence alignment at BLAST (Basic Local Alignment Search Tool) and CLUSTALW tools from DNA Data Bank of Japan (DDBJ)[43], National Center for Biological Information (NCBI)[44] and GenomeNet [45] analysis showed ≥95% sequence identity to human LH/CGR and ≥99% sequence identity to monkey LH/CGR (Additional file 4: Figure S2A and B). The two bands i.e., the upper band corresponding to full length (519/718 bp) transcript and the lower band (333/532 bp) corresponding to splice variant lacking exon 9 were detected throughout the luteal phase (Figure 1C).

The role of the splice variant lacking exon 9 is unclear as while it is observed in the human ovary and binds both LH and hCG in transfected in COS-1 cells, it could not stimulate adenylate cyclase [46]. Further, it was reported that this splice variant interacted with human follitropin receptor (hFSHR) and negatively regulated its expression by trapping them in the endoplasmic reticulum [47-49]. In the monkey CL, the steady state mRNA levels for FSHR were examined by both semi-quantitative RT-PCR and qPCR analyses throughout the luteal phase (Additional file 5: Figure S3). A primer set exon 11-11(F3-R3) designed outside the region of alternative splicing to include all splice variants of the LH/CGR and a primer set exon 9-9 (F4-R4) designed within the exon 9 to examine the expression of exon 9 containing variant. Semi-quantitative RT-PCR analysis using these primer sets amplified transcripts throughout the luteal phase, which followed the pattern of full length LH/CGR transcript expression (Figure 1C). Immunoblot analysis of LH/CGR indicated that the protein was present throughout the luteal phase including on day 1 of menses (Figure 1D). To further investigate the functionality of LH/CGR present on day 1 of menses, exogenous administration of rhLH caused significant (p < 0.05) increase in circulating P_4 _levels (0.23 ± 0.06 vs. 1.38 ± 0.26 ng/ml at 0 and 2 h post rhLH injection, respectively, Figure 1E).

### Expression and characterization of genes belonging to SFKs and their functional activation in the monkey CL throughout the luteal phase

**Figure 2:**
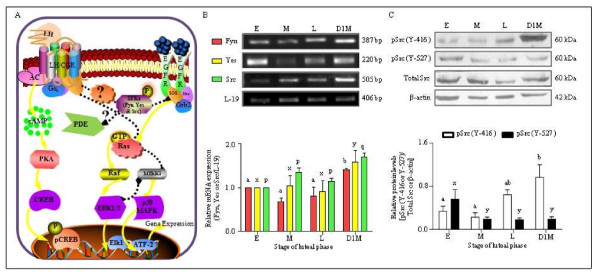
**Expression and characterization of genes belonging to SFKs and their functional activation in the monkey CL throughout the luteal phase**. (A) Diagrammatic representation of various signalling cascades and molecular players involved downstream of LH/CGR activation regulating various genes associated with steroidogenesis. Based on the review of literature the canonical pathway Gs/cAMP/protein kinase A (PKA)/pCREB and alternate signalling pathways like p38 MAPK or Erk have been illustrated in the diagram. Furthermore, recent studies describing involvement of SFKs and cAMP-PDE in modulation of hCG responsiveness and transactivation of tyrosine kinase receptors in mouse Leydig tumor cells are also represented for their possible similar role in the monkey luteal cells. (B) Semi-quantitative RT-PCR expression of genes belonging to SFKs (Fyn, Yes and Src) in the monkey CL obtained from different stages of the luteal phase. Each bar represents mean ± SEM values (n = 3 CL/stage). L-19 mRNA was used as internal control and relative expression was calculated following densitometric analysis. Bars with different letters indicate statistical significance (p < 0.05). (C) Levels of Src family of kinase protein in the monkey CL throughout the luteal phase. Immunoblot analysis was performed to determine functional activation of Src protein i.e., protein levels of active pSrc (Y-416), inactive pSrc (Y-527) and total Src in the monkey CL collected during different stages of the luteal phase. Anti-β-actin antibody (the protein loading control) probed blot is presented to indicate equal amount of protein being loaded in each lane. Densitometric analysis of immunoblots was determined and is indicated as mean ± SEM of relative amount of pSrc (Y-416/Y-527) expressed as intensity of total Src/β-actin bands for each stage of luteal phase (n = 3 CL/stage). Bars with different letters indicate statistical significance (p < 0.05).

Figure 2A shows pictorial representation of various signaling molecules many of which have been identified to be downstream of LH/CGR activation leading to gene expression changes. In addition to the well established canonical pathway and MAP kinase activation pathways, the likely cross talk between MAP kinase and EGFR activated SFK's has been depicted (Figure 2A). The role of SFKs downstream of LH/CGR activation was examined in the CL throughout the luteal phase, while the mRNA expression for Fyn, Yes and Src was present throughout the luteal phase, expression was higher in CL collected on day 1 of menses (Figure 2B). The PCR products of Fyn, Yes and Src were gel eluted, sequenced and the nucleotide sequences was ≥90% homology to human and rhesus monkey confirming the presence of SFKs transcripts in the CL. The expression of Fyn, Yes and Src was higher (p < 0.05) on day 1 of menses compared to other stages of luteal phase. The activity of Src kinase is regulated post translationally by phosphorylation of tyrosine (Y) residues in the catalytic domain i.e., phosphorylation at Y-527 would lead to inactivation, whereas phosphorylation at Y-416 causes full activation. Immunoblot analysis revealed presence of higher levels of inactive Src (pSrc Y-527) in early stage CL compared to other stages. On the other hand, activated Src (pSrc Y-416) was high in CL from late and day 1 of menses compared to CL from early and mid stage of the luteal phase (Figure 2C).

### Expression and characterization of cAMP-PDE (PDE4D) and its functional activation in the monkey CL during the luteal phase

**Figure 3:**
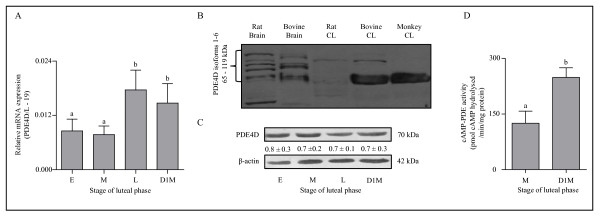
**Expression and characterization of cAMP-PDE (PDE4D) and its functional activation in the monkey CL throughout the luteal phase**. (A) The qPCR expression of PDE4D mRNA, an ovarian specific cAMP-PDE in the monkey CL obtained from different stages of the luteal phase. The relative mRNA expression at each stage is represented in each bar as mean ± SEM values (n = 3 CL/stage).Bars with different letters indicate statistical significance (p < 0.05). (B) To standardize PDE4D protein analysis conditions in the monkey CL, immunoblot analysis with anti-PDE4D was performed employing brain and CL tissue lysates from various species. (C) Levels of PDE4D protein in the monkey CL throughout the luteal phase.. Anti-β-actin antibody (the protein loading control) probed blot is presented to indicate equal loading of protein in each lane. Densitometric analysis of immunoblots was determined and relative protein levels are indicated as mean ± SEM values below individual bands. (D) Determination of cAMP-PDE activity in CL tissue lysates obtained at mid stage (M) and on day 1 of menses (D1M). Each bar represents mean ± SEM values (n = 3 CL/stage). Bars with different letters indicate statistical significance (p < 0.05).

To determine the role of PDE, mRNA and protein levels of an ovarian specific cAMP-PDE (PDE4D) were examined throughout the luteal phase. The qPCR analysis of PDE4D mRNA expression was low during early and mid luteal phase, but the expression was significantly (p < 0.05) higher at late luteal phase and day 1 of menses compared to early and mid-luteal phase (Figure 3A). Since, alternative splicing of the PDE4D mRNA results in generation of as many as 12 isoforms (PDE4D1-12), isoform specific primers were designed and semi quantitative RT-PCR analysis for PDE4D3, PDE4D5 and PDE4D6 isoforms was performed and results showing presence of all isoforms examined in the CL are presented in Additional file 6: Figure S4. Immunoblot analysis for PDE4D protein performed utilizing antibody that detects isoform PDE4D 1-6 showed presence of a single PDE4D isoform corresponding to ~70 kDa in the monkey CL, however, all six isoforms of PDE4D 1-6 were detected in rat and bovine brain tissues utilized as positive control (Figure 3B). In the bovine CL, this anti-PDE4D antibody detected two isoforms corresponding to ~70 kDa and ~95 kDa isoforms whereas, in the rat CL probing with the same antibody detected a single band corresponding to ~95 kDa (Figure 3B). The PDE4D isoform was detectable throughout the luteal phase and the results of representative blot are shown in Figure 3C.

The role of PDE4D depends on its functional activity, and analysis of cAMP-PDE activity indicated a significantly (p < 0.05) higher activity in CL tissue collected from monkeys on day 1 of menses compared to the mid stage CL (248.76 ± 26.12 vs. 125.14 ± 32.58 pmol cAMP hydrolysed/min/mg protein in D1M and MCL, respectively; Figure 3D). Since, LH mediates its action primarily by activating classical cAMP/PKA/CREB pathway, activation of SFKs or in turn PDE4D might modulate one of these components to regulate LH signaling. The PDE4D hydrolyzes cAMP to 5'AMP and thus play a critical role in the regulation of intracellular cAMP levels. It was of interest to examine the cAMP content in CL tissues throughout the luteal phase. The cAMP content was high in mid stage compared to CL from late stage and on day 1 of menses and the data is presented in Additional file 7: Figure S5A, The findings of higher cAMP-PDE activity together with higher pSrc (Y-416) levels strongly suggest involvement of SFKs and PDE in modulation of LH actions leading to loss of function and eventually regression of CL at the end of non-fertile cycle.

### Expression and characterization of LH/CGR and genes belonging to SFKs in the monkey CL during PGF_2α_-induced luteolysis

**Figure 4:**
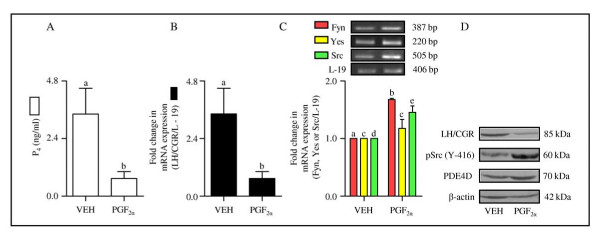
**Expression and characterization of LH/CGR and genes belonging to SFKs in the monkey CL during PGF_2α_-induced luteolysis**. (A) Circulating P_4 _levels 24 h after VEH/PGF_2α _treatment. Each bar represents mean ± SEM values (n = 3 animals/treatment). (B) The fold change in qPCR expression for LH/CGR mRNA in the CL 24 h post VEH/PGF_2α _treatment. Each bar represents mean ± SEM values (n = 3 animals/treatment). (C) Semi-quantitative RT-PCR expression of genes belonging to SFKs (Fyn, Yes and Src) in the monkey CL obtained 24 h post VEH/PGF_2α _treatment. L-19 mRNA was used as internal control and the relative expression was calculated following densitometric analysis. Each bar represents mean ± SEM values (n = 3 animals/treatment). (D) Levels of LH/CGR, active pSrc (Y-416) and PDE4D protein in the monkey CL obtained 24 h post VEH/PGF_2α _treatment. Anti-β-actin antibody (the protein loading control) probed blot is presented to indicate equal loading of protein in each lane.

Expression of LH/CGR and SFKs was examined in the CL collected during PGF_2α_-induced luteolysis. Circulating P_4 _levels were 3.43 ± 1.06 ng/ml post VEH treatment, but after PGF_2α _treatment the levels were 0.73 ± 0.28 ng/ml at 24 h (Figure 4A). Expression of LH/CGR mRNA by qPCR analysis showed 4 fold decrease (p < 0.05) in LH/CGR mRNA in the CL from PGF_2α _treated monkeys (Figure 4B). Semi-quantitative RT-PCR expression analysis indicated increased (p < 0.05) expression of Fyn and Src mRNAs in CL from PGF_2α _treated monkeys (Figure 4C). The LH/CGR protein expression was low (p < 0.05) and activated Src (pSrc Y-416) expression was high (p < 0.05) in CL tissues collected from PGF_2α _treated monkeys (Figure 4D).

### Expression and characterization of LH/CGR, genes belonging to SFKs and PDE4D in the CL during LH secretion inhibition and LH replacement

**Figure 5:**
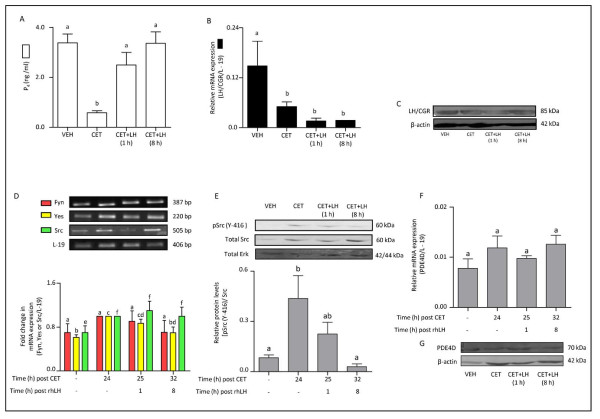
**Expression and characterization of LH/CGR, genes belonging to SFKs and PDE4D in the monkey CL following LH secretion inhibition and LH replacement**. (A) Circulating P_4 _levels following different treatments. Each bar represents mean ± SEM values. For comparison among various treatment groups, one-way ANOVA analysis was performed and bars with different letters indicate statistical significance (p < 0.05). (B) The qPCR expression for LH/CGR mRNA in the CL following different treatments. Each bar represents mean ± SEM values (n = 3 animals/treatment). For comparison among various treatment groups, one-way ANOVA analysis was performed and bars with different letters indicate statistical significance (p < 0.05). (C) Levels of LH/CGR protein in the monkey CL post CET-induced luteolysis and rescue of CL function at 1 or 8 h post CET + rhLH treatment. Anti-β-actin antibody (the protein loading control) probed blot is presented to indicate equal loading of protein in each lane. (D) Semi-quantitative RT-PCR expression of genes belonging to SFKs (Fyn, Yes and Src) in the monkey CL obtained following different treatments. L-19 mRNA was used as internal control and the fold change in mRNA expression was calculated following densitometric analysis. Each bar represents mean ± SEM values (n = 3 animals/treatment). For comparison among various treatment groups, one-way ANOVA analysis was performed and bars with different letters indicate statistical significance (p < 0.05). (E) Immunoblot analysis was performed to examine functional activation of Src protein i.e., protein levels of active pSrc (Y-416) and total Src in the monkey CL collected 24 h post VEH/CET treatment and at 1 or 8 h post CET + rhLH treatments. Protein lysates (100-200 μg) prepared from CL were resolved on 10% SDS PAGE, transferred on to PVDF membrane and immunoblot analysis was performed using antibodies raised against pSrc (Y-416), total Src and total Erk. A representative immunoblot blot for each of the protein antibody probed is shown along with the size of the protein. Anti-Erk antibody (the protein loading control) probed blot is presented to indicate equal loading of protein in each lane. Densitometric analysis of immunoblots was determined and the values are indicated as mean ± SEM of relative amount of pSrc (Y-416) expressed as intensity of total Src bands in each group (n = 3 animals/treatment group). For comparison among various treatment groups, one-way ANOVA analysis was performed and bars with different letters indicate statistical significance (p < 0.05). (F) The qPCR expression of PDE4D mRNA, in the CL obtained from monkeys subjected to different treatments. Each bar represents mean ± SEM values (n = 3 animals/treatment). For comparison among various treatment groups, one-way ANOVA analysis was performed (represented by letter on each bar) and the data across treatment groups was not significantly different (p > 0.05). (G) Immunoblot analysis was performed to examine PDE4D protein levels in the CL collected from monkeys subjected to different treatments. A representative immunoblot probed along with anti-β-actin antibody is presented to indicate equal loading of protein in each lane.

To examine the effects of LH secretion inhibition and its subsequent replacement on regulation of SFKs and PDE activation, monkeys subjected to LH secretion inhibition by a GnRH receptor antagonist, CET treatment and replacement of LH in CET-treated monkeys were employed.On day 8 of luteal phase monkeys were administered VEH/CET and CL was collected at the end of 24 h treatment (n = 3/VEH or CET treatment) or monkeys were further treated with PBS or rhLH 24 h post VEH or CET treatment and CL collected at the end of 1 or 8 h PBS/rhLH treatment. Circulating P_4 _levels were 3.38 ± 0.35 ng/ml before CET treatment and decreased significantly to reach 0.58 ± 0.08 ng/ml at 24 h after initiation of CET treatment (p < 0.05; Figure 5A). Administration of rhLH in CET-treated monkeys resulted in brisk increase in P_4 _levels within 8 h (p < 0.05; Figure 5A). Expression of LH/CGR mRNA by qPCR analysis indicated ~3 fold decrease post CET treatment, which did not change (p < 0.05) at 1 and 8 h post LH treatment in CET-treated monkeys (Figure 5B). Immunoblot analysis for LH/CGR protein in CL tissue lysates did not show detectable changes following CET-induced luteolysis or rescue of CL function by LH replacement (Figure 5C). The cAMP levels were low post CET treatment, but increased upon LH replacement (Additional file 7: Figure S5B). Semi-quantitative RT-PCR analysis indicated Yes expression was low in CL from VEH treated monkeys increased (P < 0.05) in CL from 24 h CET and CET + LH (1 h) treated monkeys. Src mRNA expression was higher after CET and CET + LH treated monkeys (Figure 5D), while, expression of Fyn was not statistically significant after CET and LH treatments (Figure 5D). The immunoblot analysis showed higher activated Src (pSrc Y-416) in CL tissues from CET-treated monkeys, while LH replacement was able to reverse pSrc (Y-416) activation (p < 0.05; Figure 5E). The qPCR analysis for PDE4D mRNA and immunoblot analysis for PDE4D protein indicated no significant change post CET treatment or LH replacement (Figure 5F, G).

### Expression and characterization of LH/CGR and genes belonging to SFKs in the monkey CL during simulated early pregnancy condition

**Figure 6:**
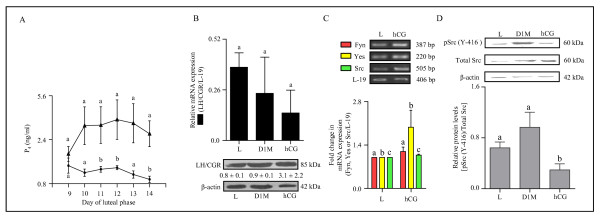
**Expression and characterization of LH/CGR and genes belonging to SFKs in the monkey CL during simulated early pregnancy condition**. (A) The graph illustrates circulating mean P_4 _levels during different days of the luteal phase without (solid circles) and with hCG (solid triangle) treatment. Each point represents mean ± SEM values (n = 3 animals/control or hCG treatment). Bars with different letters indicate statistical significance (p < 0.05). (B) The qPCR expression for LH/CGR mRNA (upper panel) and the levels of LH/CGR protein (lower panel) in the CL from monkeys collected on day 14 without treatment i.e., late CL (L), on day 1 of menses (D1M) and on day 14 following hCG treatment from day 9-13 of luteal phase (hCG). Each bar in the upper panel represents mean ± SEM values (n = 3 animals/treatment). For comparison among various groups, one-way ANOVA analysis was performed and the data across groups was not significantly different (p > 0.05). The representative immunoblots probed with anti-LH/CGR antibody and anti-β-actin antibody are shown in the lower panel. Densitometric analysis (mean ± SEM for n = 3) of immunoblots was performed and the protein levels are indicated below respective bands. L19 mRNA was used as internal control for qPCR, while β-actin protein level was used as loading control. (C) Semi-quantitative RT-PCR expression of genes belonging to SFKs (Fyn, Yes and Src) in the CL collected from monkeys on day 14 of luteal phase that received hCG treatment to stimulate early pregnancy (hCG) or without treatment Late (L). Each bar represents mean ± SEM values (n = 3 animals/control or hCG treatment). L-19 mRNA was used as internal control and fold change in mRNA expression was calculated following densitometric analysis. (D) Immunoblot analysis was performed to determine functional activation of Src protein i.e., protein levels of active pSrc (Y-416) and total Src in the CL collected from monkeys on late luteal phase (L), on day 1 of menses (D1M) and on day 14 of luteal phase following hCG treatment on day 9-13 of luteal phase (hCG). A representative immunoblot for each of the protein antibody probed is shown along with the size of the protein. Anti-β-actin antibody (the protein loading control) probed blot is presented to indicate equal loading of protein in each lane. Densitometric analysis of immunoblots was determined and level of pSrc (Y-416) is expressed as mean ± SEM relative to the intensity of total Src/β-actin. Individual bars with different letters are significantly different (p < 0.05).

During conception cycles, the function of CL is transiently rescued by the CG secreted from the placental trophoblast. The exogenous administration of incremental hCG during non fertile cycle to simulate early pregnancy has been standardized in the monkey. As can be seen from Figure 6A, circulating P_4 _levels increased significantly with hCG treatment indicative of increased CL function (p < 0.05; Figure 6A). However, analysis of LH/CGR mRNA and protein levels post hCG treatment did not show a significant change compared to CL from monkeys not receiving hCG treatment and from day 1 of menses (p > 0.05; Figure 6B). Expression of Fyn, Yes and Src mRNAs by semi-quantitative RT-PCR analysis was unaffected (p > 0.05) by hCG treatment, (Figure 6C). However, activated pSrc (Y-416) levels were significantly low post hCG treatment compared to levels in CL from monkeys collected at the late stage and on day 1 of menses (p < 0.05; Figure 6D).

### Expression and characterization of genes associated with steroidogenesis/cholesterol trafficking (HMGR and SR-B1) and transcription factors (LRH-1 and SF-1) downstream of LH/CGR signaling

**Figure 7:**
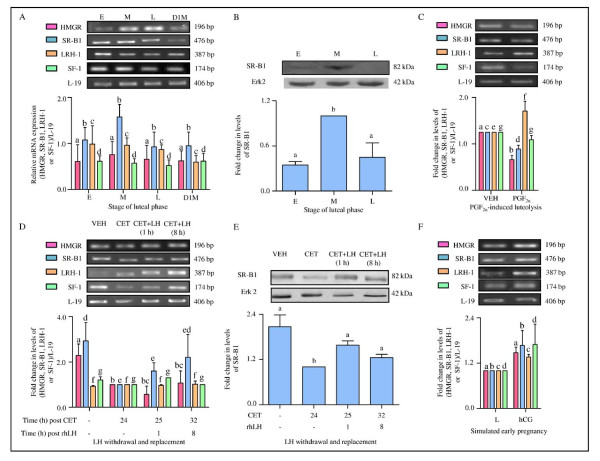
**Expression and characterization of genes associated with steroidogenesis/cholesterol trafficking (HMGR and SR-B1) and transcription factors (LRH-1 and SF-1) downstream of LH-LH/CGR activation**. (A) Semi-quantitative RT-PCR expression of mRNA encoded by HMGR, SR-B1, LRH-1 and SF-1 genes in the CL obtained from monkeys during different stages of the luteal phase (E: early, M: mid, L: late and D1M: day 1 of menses). Each bar represents mean ± SEM values of three independent experiments. L-19 mRNA was used as internal control and relative expression was calculated following densitometric analysis. (B) Immunoblot analysis was performed to determine SR-B1 protein levels in the monkey CL collected during different stages of the luteal phase. A representative immunoblot blot along with blot probed against anti-Erk2 antibody (as protein loading control) is presented. Densitometric analysis of immunoblots was determined and is shown as mean ± SEM of SR-B1 levels relative to the intensity of Erk2 band in each stage (n = 3 animals/stage). Individual bars with different letters are significantly different (p < 0.05). (C) Semi-quantitative RT-PCR expression of HMGR, SR-B1, LRH-1 and SF-1 mRNAs in the CL obtained from monkeys 24 h after VEH/PGF_2α _treatment. Each bar represents mean ± SEM values of three independent experiments. L-19 mRNA was used as internal control and the fold change in levels of mRNA for each gene was calculated by densitometric analysis. (D) Semi-quantitative RT-PCR expression of HMGR, SR-B1, LRH-1 and SF-1 mRNAs in the CL obtained from monkeys post CET-induced luteolysis and rescue of CL function. Each bar represents mean ± SEM values of three independent experiments. L-19 mRNA was used as internal control and the fold change in levels of mRNA for each gene was calculated by densitometric analysis. (E) Immunoblot analysis of SR-B1 levels in the CL collected from monkeys post CET-induced luteolysis and rescue of CL function. A representative immunoblot along with blot probed against anti-Erk2 antibody (as protein loading control) is presented. Densitometric analysis of immunoblots is shown as mean ± SEM of SR-B1 levels relative to the intensity of Erk2 bands (n = 3 animals/treatment). Individual bars with different letters are significantly different (p < 0.05). (F) Semi-quantitative RT-PCR expression of HMGR, SR-B1, LRH-1 and SF-1 mRNAs in the CL collected from monkeys on day 14 of luteal phase without treatment (Late-L) or with hCG treatment to stimulate early pregnancy (hCG). Each bar represents mean ± SEM values of three independent experiments. L-19 mRNA was used as internal control and fold change in levels of mRNA for each gene was calculated by densitometric analysis.

The results from the previous experiments indicated that the activated SFKs and cAMP-PDE modulate the molecules downstream of LH/CGR signaling. Another important aspect of LH/CGR signaling is the regulation of cholesterol economy of luteal cells for optimal P_4 _biosynthesis and in turn maintenance of CL function. To examine the role of cholesterol trafficking in the P_4 _biosynthesis some of the molecules that regulate cholesterol economy of luteal cells were examined by semi-quantitative RT-PCR and immunoblot analysis. The mRNA expressions of HMGR, SR-B1, LRH-1 and SF-1 were not significantly (p > 0.05) different at different stages examined (Figure 7A). The protein levels of SR-B1 were significantly (p < 0.05) higher in mid stage CL compared to CL from early and late stage luteal phase (Figure 7B). In CL of monkeys treated with PGF_2α_, mRNA expressions of HMGR and SR-B1 were lower (p < 0.05) compared to VEH treated monkeys, while expression of LRH-1 increased and SF-1 expression did not change (p > 0.05; Figure 7C). Following CET treatment, HMGR expression decreased (p < 0.05) at 24 h post treatment, remained lower in CL from 1 h LH treatment monkeys, but recovered in CL of monkeys that received LH treatment for 8 h (Figure 7D). Expression of SR-B1 in CL was lower (p < 0.05) 24 h post CET treatment, but returned to pre CET treatment levels 8 h after LH replacement (Figure 7D). Changes in LRH-1 and SF-1 expressions were not seen in CL of monkeys receiving CET or CET + LH (p > 0.05; Figure 7D). Expression of SR-B1 protein declined 24 h post CET treatment (p < 0.05; Figure 7E). In CL of monkeys treated with hCG, mRNA expressions of HMGR, SR-B1, LRH-1 and SF-1 were not statistically higher (p > 0.05; Figure 7F).

### A model depicting the overall results showing various molecular players involved downstream of LH/CGR activation resulting in the regulation of expression of genes associated with steroidogenesis in the monkey CL

**Figure 8:**
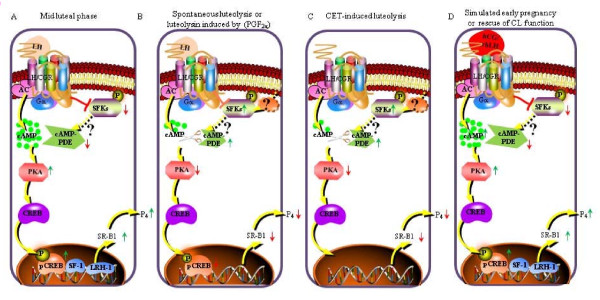
**A diagrammatic representation of the overall results showing various signalling molecules modulated downstream of LH/CGR activation regulating genes associated with steroidogenesis during different functional status in the monkey CL**. (A) The diagram describes activation of the well characterized LH-mediated canonical pathway LH/CGR/Gα_s_/AC/cAMP/PKA/(pCREB, SF-1/LRH-1) in the monkey CL, during mid luteal phase (when the CL function is high) leading to increase in SR-B1 gene expression (both mRNA and protein levels) and in turn higher serum P_4 _levels. Further, it also illustrates prevention of SFKs activation and lower cAMP-PDE activity in presence of LH during mid stage of luteal phase, inturn avoiding modulation of LH/CG responsiveness by the molecular modulators [pSrc (Y-416) and PDE4D]. (B) The diagram demonstrates decrease in LH-mediated inhibition of SFKs activation and further increase in both SFKs phosphorylation and cAMP-PDE activity in the monkey CL during spontaneous luteolysis or PGF_2α_-induced luteolysis (CL function is low) leading to modulation of LH/CG responsiveness in turn resulting in lower circulating P_4 _levels. (C) To determine the role of LH in regulation of SFKs activity, LH withdrawal using GnRH-R antagonist (CET) was performed. The diagram describes the elimination of LH-mediated activation of canonical signalling and increase in both SFKs phosphorylation and cAMP-PDE activity in the monkey CL during CET-induced luteolysis (CL function is low) leading to decrease in SR-B1 gene expression and in turn lower circulating P_4 _levels. (D) To determine the role of LH in regulation of SFKs activity, simulation of early pregnancy condition was developed and LH replacement in CET treated animals to rescue CL function was performed. The diagram illustrates recurrence of LH/CG-mediated cAMP/PKA/pCREB signalling and suppression of SFKs activation leading to lower cAMP-PDE activity, higher SR-B1 gene expression and in turn higher circulating P_4 _levels.

In the monkey CL during mid luteal phase (Figure 8A) or during rescue of CL function by LH/CG (Figure 8D), the SFKs or cAMP-PDE activities are kept in check allowing activation of cAMP/PKA/(pCREB, SF-1/LRH-1) canonical pathway causing increase in expression of SR-B1 that results in higher circulating P_4_. Whereas, spontaneous luteolysis (Figure 8B) or induced luteolysis by PGF_2α_/CET (Figure 8C) cause withdrawal of inhibition on activation of SFKs resulting in higher cAMP-PDE activity leading to attenuation of LH/CG responsiveness, decrease in SR-B1 expression and lowered circulating P_4_.

## Discussion

In higher primates, the mechanisms responsible for luteal regression that occurs at the end of non-fertile cycle are poorly understood. In the present studies, efforts were made to explain the dichotomy of LH/CG control of luteal function during non-fertile/fertile cycles in the monkey. We hypothesized that the signaling pathways downstream of LH/CGR may play a crucial role in modulating the LH/CG actions and in turn CL function. The results of studies carried out employing various model systems clearly suggest involvement of SFKs and cAMP-PDE in the decreased responsiveness of LH/CGR activated signaling during luteolysis. Furthermore, the results emphasize the importance of SR-B1, a membrane receptor protein, for trafficking of cholesterol to be an additional regulatory step influencing steroidogenesis in luteal cells.

In the present study, LH/CGR expression was observed to be high at the late luteal phase and the expression was detectable even on day 1 of menses in contrast to non detectable expression on day 1 of menses reported earlier by others [42,50]. Cameron et al., 1982 [51] reported that the decline in P_4 _levels seen during late luteal phase was not accompanied by appreciable decline in number and affinity of LH/CGR. The results from this study further extend that observation, since the regressing CL continues to maintain responsiveness to exogenous LH/CG stimulation. To rule out the possibility that the changes in differentially expressed variants of LH/CGR as previously reported in the monkey [42], human [52], rat [53] and the bovine [54], do not contribute to the onset of luteolysis, presence of multiple splice variants was examined employing primers designed around the hinge region of LH/CGR gene. Several studies have reported transcript variants of LH/CGR involving mutations in exon 7 and/or absence of exon 10 leading to loss of receptor expression to the plasma membrane or loss of LH binding, but not CG binding to receptor [55-57]. In the present study, expression of splice variant lacking exon 9 was characterized and this variant has previously been reported in humans [46] and rats [58]. Although the presence of full-length LH/CGR transcript and the splice variant lacking exon 9 could be established in the monkey CL, we could not find the differential expression of these variants as reported for human luteal cells, in which the truncated variant has been suggested to modulate the cell surface expression and activity of full-length LH/CGR [59]. The role of monkey LH/CGR splice variant lacking exon 9 is not clear, but as reported previously in human luteal cells, activation of the splice variant may not lead to increased adenylate cyclise activity [46] or may negatively modulate expression of wild type gonadotropin receptors by trapping them in the ER [47-49]. In the monkey, sequestration of gonadotropin receptors by the exon 9 lacking variant was ruled out since full length LH/CGR expression was high during the late luteal phase as shown in Figure 1B, D while the FSHR expression was low through most part of the luteal phase as shown in Additional file 5: Figure S3 indicating lack of interaction between LH/CGR transcript variant and LH/CGR or FSHR. The findings of LH/CGR expression and analysis of its transcript variants taken together suggest that the cause for decline in CL function does not appear to be due to decrease in LH/CGR expression or changes in expression pattern of LH/CGR splice variants. Surprisingly, mRNA expression of LH/CGR post LH inhibition and replacement showed lower expression but it was not reflected in the changes in protein levels. One explanation for this discrepancy is that mRNA levels are low and fluctuating in CL of VEH treated animals. Also, the antibody employed against LH/CGR detects one isoform that does not appear change irrespective of treatments. Moreover, it is possible that transcriptional changes may not be reflected in the translational changes at the time points examined.

The review of literature on effects of LH/CG suggests regulation of various functions of luteal and other steroidogenic cells involve activation of multiple signaling pathways [14,60,61]. Although LH mediates its action by Gs/cAMP/PKA/CREB canonical pathway, recently it has been reported that activation of LH/CGR also results in activation of other G proteins [11,12] and receptors involving tyrosine phosphorylation leading to activation of Ras/ERK1/2 cascade [16,20,21,62,63]. Activation of ERK cascade may involve other signaling molecules such as SFKs, β-arrestins, PKA, Ras, Shc and the EGFR as intermediates in the pathway [64-69]. Studies carried out employing inhibitors and dominant negative constructs of SFKs have shown that many of these molecules interact with the canonical LH/CGR activated signaling pathway resulting in altered responsiveness to steroidogenesis in ovarian cells [19,70,71], MA-10 tumor Leydig cells [18,72,73] and adrenal cells [74,75]. In the present study, expression of genes belonging to SFKs confirm low levels of activated Src during high steroidogenic state or increased activated levels of Src during low steroidogenic state of the CL. The interaction between the LH/CGR and SFKs can possibly either be a direct [17,76] or indirect involving Pyk2 [77] or β-arrestins [66,78] to keep Src activation in check. Thus, increased Src activation observed during low P_4 _secretion may be due to reduced responsiveness to LH since cAMP-PDE activity involving Ras activation may be affected as observed in MA-10 and ovarian theca cells [18,19,73]. The role of PDE in the regulation of endocrine cell functions has been well documented [79-83]. In the present study, presence of few PDE4D isoforms in the CL tissue also as reported in other cells was confirmed [84-87]. Initial attempts to correlate increased PDE activity with Src activation was not successful as it was difficult to determine the direct/indirect interactions in the monkey CL as reported for other cell types [88-93]. Nonetheless, the results obtained employing different models systems in the present study and as hypothesized by other investigators, it can be suggested that modulation of LH/CGR signaling by Src might involve a role for PDE.

Several studies suggest important role for regulation of StAR expression by LH in primates [94]. In species such as bovines, StAR expression appears to be central to regulation of CL function and further, it may be viewed that steroidogenesis is critically dependent on factors that regulate StAR expression [95]. However, the results from the present study, suggest that cholesterol acquisition by luteal cells involving receptor proteins such as SR-B1 appears to be also equally important for regulation of CL function in monkeys. SR-B1 mRNA and protein levels corroborate well with the steroidogenic status of the CL as reported previously for the ovary [96,97], adrenal [31,98] and Leydig cells [99,100]. Studies by others have also suggested that SR-B1 to be a key LH regulated gene modulating steroidogenic activity as observed in the present study [27,96]. Moreover, presence of orphan nuclear transcription factors such as LRH-1 and SF-1 expression in the monkey CL may suggest transcriptional regulation of SR-B1 expression as observed in the bovine CL [32]. In summary, based on the results of the present study, at least four possible scenarios represented pictorially in Figure 8 can be thought of to explain the regulation of CL function during different functional status. They are: 1) CL during maximal steroidogenic state (mid luteal phase) and consequently high circulating P_4 _levels. Upon activation of Gs/cAMP/PKA/CREB pathway by LH leads to increased expression of genes such as SR-B1. Further, presence of the decreased activated pSrc (Tyr-416) level indicates LH signaling to be inhibitory to Src activation and lowered cAMP-PDE activity. 2) CL during spontaneous luteolysis and/or following PGF_2α_-induced luteolysis is concordant with lowered circulating P_4 _levels. The LH canonical signaling pathway is modulated by increased activated pSrc (Tyr-416) levels and cAMP-PDE activity leading to lowered cAMP levels, PKA activity and pCREB levels which corroborate well with decreased expression of SR-B1. 3) CL during decreased luteal function due to inhibition of circulating LH by CET treatment leading to low circulating P_4 _levels. The inhibition of LH canonical signaling pathway leads to increase in activated pSrc (Tyr-416) levels and cAMP-PDE activity with concomitant decrease in SR-B1 expression, and 4) CL during simulated early pregnancy condition and after LH replacement in the CET-treated monkeys causing higher or rescued function of CL resulting in higher circulating P_4 _levels. The Gs/cAMP/PKA/CREB pathway is robust with higher cAMP levels, increased PKA activity and increased pCREB expression consequently decreased activated pSrc (Tyr-416) and cAMP-PDE levels, finally leading to higher SR-B1 expression.

## Conclusions

The results indicate participation of activated Src [pSrc (Tyr-416)] and increased activity of cAMP-PDE during spontaneous luteolysis, while LH/hCG treatment caused decreased activation of Src and cAMP-PDE activity concomitant with increased secretion of P_4 _in the CL.

## Competing interests

The authors declare that they have no competing interests.

## Authors' contributions

KBS & RM participated in designing, conducting experiments, analysis of results and preparation of manuscript. AK participated in analysis of data and preparation of manuscript. All authors read and approved the final manuscript.

## Supplementary Material

Additional file 1 **Table S1: List of primers employed for semi-quantitative RT-PCR analysis**. Click here for file

Additional file 2 **Table S2: List of primers employed for qPCR analysis**. Click here for file

Additional file 3 **Figure S1: Schematic representation of LH/CGR gene depicting exons, number of nucleotides in each exon region and various structural domains formed by each group of exons**. The arrows indicate the multiple primer sets designed around the alternatively spliced regions to detect various splice variants of LH/CGR by RT-PCR analysis. The positions of forward primers, F1 and F2 on exon 7 and 5, while position of their respective reverse primers, R1 and R2 on exon 11 are represented. The positions of two other primer sets (F3-R3 and F4-R4) spanning the extreme 3' end of exon 11 region and within the exon 9 region are also represented. The details of splice variants of LH/CGR reported in literature and the calculated PCR product size for each of the possible splice variants detected employing multiple primer sets F1-R1 and F2-R2 in the present study are shown. Click here for file

Additional file 4 **Figure S2: The blast analysis of nucleotide sequences obtained after sequencing the upper and lower bands obtained using multiple primer pair set F2-R2 (exon5-11)**. Shown here is the multiple sequence alignment of the PCR product sequence [(A) LH/CGR upper band (718 bp) and (B) lower band (532 bp)] compared with Gen Bank database sequence of human and monkey species depicting the sequence identity (*). Click here for file

Additional file 5 **Figure S3: (A) Schematic representation of FSHR gene depicting exons, various structural domains formed by exons and number of nucleotides in each exon region**. The arrows indicate the primer set representing position of forward primers (F1&F2) and reverse primers (R1&R2) designed around the extracellular domain and hinge regions to detect FSHR mRNA transcripts (sizes 680 and 151 bp). (B) Semi-quantitative RT-PCR analysis to determine FSHR mRNA expression in the monkey CL during different stages of the luteal phase (E: early, M: mid, L: late and D1M: day 1 of menses). L-19 mRNA was used as internal control and the relative expression was calculated following densitometric analysis. Each bar represents mean ± SEM values (n = 3 CL/stage). No significant differences (p > 0.05; denoted by the same letter on individual bars) in expression was seen throughout the luteal phase. (C) The qPCR analysis for FSHR mRNA expression in the monkey CL during different stages of the luteal phase. The fold change in mRNA expression at each stage CL compared to early stage is represented in each bar as mean ± SEM values (n = 3 CL/stage). FSHR expression was high at mid luteal phase, but the expressions at late and D1M were low. Individual bars with different letters are significantly different (p < 0.05). Click here for file

Additional file 6 **Figure S4: Semi-quantitative RT-PCR analysis of PDE4D isoforms [PDE4D3 (A), PDE4D5 (B) and PDE4D6 (C)] was performed in CL tissue collected from monkeys during different stages of the luteal phase**. The expression of housekeeping gene, L19, was used as the internal control. Shown here is a representative gel picture of PDE4D isoforms and L-19 PCR amplification products. Click here for file

Additional file 7 **Figure S5: (A) Tissue cAMP levels in the monkey CL collected during different stages of the luteal phase**. Individual bars with different letters indicate statistical significance (p < 0.05). (B) Tissue cAMP levels from animals treated with Vehicle (VEH), CET and CET + LH (LH replacement). Individual bars with different letters indicate statistical significance (p < 0.05). Click here for file
