# Supplementary material for: Involvement of Src family of kinases and cAMP phosphodiesterase in the luteinizing hormone/chorionic gonadotropin receptor-mediated signaling in the corpus luteum of monkey
Source: Reprod Biol Endocrinol. 2012 Mar 29;10:25. doi: 10.1186/1477-7827-10-25 (PMC3353251; doi:10.1186/1477-7827-10-25)
Supplement: Additional file 1 — Table S1: List of primers employed for semi-quantitative RT-PCR analysis. [file 1477-7827-10-25-S1.PDF]

Table S1: List of primers employed for semi-quantitative RT-PCR analysis

Sl. No.	Gene Name	Primer Sequence (5' to 3')	Annealing Temp (ºC)	No. of cycles	Product size (bp)
1	L-19	F-GAAATCGCCAATGCCAACTC<br>R-TCTTAGACCTGCGAGCCTCA	58.0	24	406
2	LH/CGR Exon 5-11	F-CATCTGTAACACGGGCATCAGAA<br>R-CAGCCAAATCAGGACCCTAA	67.0	32	718 and 532
3	LH/CGR Exon 7-11	F-GGGACGACACTGATTTCACT<br>R-CAGCCAAATCAGGACCCTAA	60.0	30	519 and 333
4	LH/CGR Exon 11-11	F-GTGTCAGCAATTACATGAAGGTC<br>R-GTGAAGCCATTTTTGCAGTTGG	54.0	27	494
5	LH/CGR Exon 9-9	F-AAGCACAGCAGTGGCTGGGG<br>R-TGCAGGCCCTGCCGAGCTAT	68.0	28	144
6	Fyn	F-GGGCTGTGTGCAATGTAAGG<br>R-CCAGTTGTCAAGGAGCGG	62.0	33	387
7	Yes	F-GGTTGATATGGCTGCTCAG<br>R-ATACAGTGCAGCTTCAGGAGC	62.0	33	220
8	Src	F-CTGGCCATCCGGTACAGAA<br>R-GCCCTCCATGACCTTTGG	60.0	32	505
9	HMGR	F-TGATTGGAGTTGGTACCATG<br>R-ATGGCTGAGCTGCCAAAT	63.0	32	196
10	SR-B1	F-GAGATCATGTGGGGCTA<br>R-AACCTGCAGGTGCTGAC	58.0	30	475
11	SR-B1	F-TCATCAAGCAGCAGGT<br>R-ATGGCCTCCTTATCCT	50.0	24	1357
12	LRH-1	F-CCCAAAGTGGAGACGGAAG<br>R-GGATCACCTGAGACATGGCT	63.0	30	387
13	SF- 1	F-CGCGGGCATGGACTAT<br>R-CTCTCGGTGCACGTGTA	60.0	30	174
14	FSHR	F-CTCACCAAGCTTCGAGTCAT<br>R-CAGTTTGCAAAGGCACAGC	64.0	32	680
16	PDE4D3	F-GCCTCTGAGGAAACACTACA<br>R-CAGGGGGAGGCTGGTT	60.0	36	186
17	PDE4D5	F-ACAGACGTCATACAGCCCTTGAGG<br>R-GGGAGAGCTGTCAAGGAAGTTCCA	68.0	36	234
18	PDE4D6	F-ACCAGCTCTGACTTCTCGTGG<br>R-CTTGATTTGGCTCCTGTAGACGG	62.0	36	116
