# Supplementary material for: Involvement of Src family of kinases and cAMP phosphodiesterase in the luteinizing hormone/chorionic gonadotropin receptor-mediated signaling in the corpus luteum of monkey
Source: Reprod Biol Endocrinol. 2012 Mar 29;10:25. doi: 10.1186/1477-7827-10-25 (PMC3353251; doi:10.1186/1477-7827-10-25)
Supplement: Additional file 2 — Table S2: List of primers employed for qPCR analysis. [file 1477-7827-10-25-S2.PDF]

Table S2: List of primers employed for qPCR analysis

Sl. No.	Gene Name	Primer Sequence (5' to 3')	Annealing Temp (ºC)	Product size (bp)
1	L19	F: CCACATGTATCACAGCCTGTAC	66.0	151
		R: CTTGGTCTTAGACCTGCGG		
2	LH/CGR	F: ATGCCATAGACTGGCAGACAGG	66.0	129
		R:TAGCATAGGTGATGGTGTGCC		
3	PDE4D	F: CCGGAACTCCTCCATTGCC	65.0	148
		R:GGGTGATCTTTTGCTAGGTGCTC		
4	FSHR	F: GTGCATTCAATGGAACCCAAC	66.0	151
		R: AAGCCATAGCTAGGCAGGGA		
