# Supplementary material for: Involvement of Src family of kinases and cAMP phosphodiesterase in the luteinizing hormone/chorionic gonadotropin receptor-mediated signaling in the corpus luteum of monkey
Source: Reprod Biol Endocrinol. 2012 Mar 29;10:25. doi: 10.1186/1477-7827-10-25 (PMC3353251; doi:10.1186/1477-7827-10-25)
Supplement: Additional file 3 — Figure S1: Schematic representation of LH/CGR gene depicting exons, number of nucleotides in each exon region and various structural domains formed by each group of exons. The arrows indicate the multiple primer sets designed around the alternatively spliced regions to detect various splice variants of LH/CGR by RT-PCR analysis. The positions of forward primers, F1 and F2 on exon 7 and 5, while position of their respective reverse primers, R1 and R2 on exon 11 are represented. The positions of two other primer sets (F3-R3 and F4-R4) spanning the extreme 3' end of exon 11 region and within the exon 9 region are also represented. The details of splice variants of LH/CGR reported in literature and the calculated PCR product size for each of the possible splice variants detected employing multiple primer sets F1-R1 and F2-R2 in the present study are shown. [file 1477-7827-10-25-S3.PDF]

Figure S1: Schematic representation of LH/CGR gene depicting exon regions, number of nucleotides and structural domains

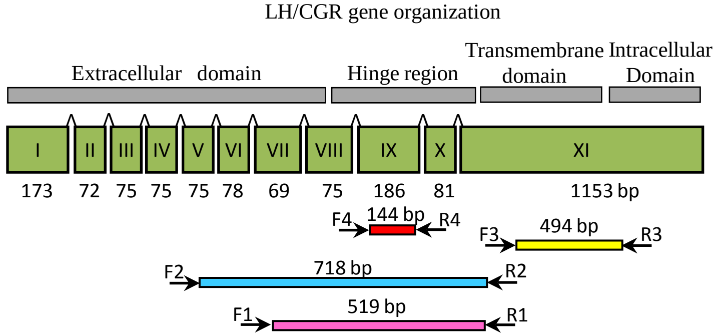

Transcript	Exon 7–11 (F1 – R1)	Exon 5–11 (F2 – R2)
Full length	519 bp	718 bp
Lacking Exon 8	444 bp	643 bp
Lacking Exon 9	333 bp	532 bp
Lacking Exon 10	438 bp	637 bp
Lacking Exon 8 and Exon10	363 bp	562 bp
