# Supplementary material for: Involvement of Src family of kinases and cAMP phosphodiesterase in the luteinizing hormone/chorionic gonadotropin receptor-mediated signaling in the corpus luteum of monkey
Source: Reprod Biol Endocrinol. 2012 Mar 29;10:25. doi: 10.1186/1477-7827-10-25 (PMC3353251; doi:10.1186/1477-7827-10-25)
Supplement: Additional file 4 — Figure S2: The blast analysis of nucleotide sequences obtained after sequencing the upper and lower bands obtained using multiple primer pair set F2-R2 (exon5-11). Shown here is the multiple sequence alignment of the PCR product sequence [(A) LH/CGR upper band (718 bp) and (B) lower band (532 bp)] compared with Gen Bank database sequence of human and monkey species depicting the sequence identity (*). [file 1477-7827-10-25-S4.PDF]

Figure S2: (A) Sequencing results for LH/CGR upper band (718 bp) obtained using multiple primer pair F2-R2 (exon 5-11)

(A) LH/CGR Upper band

```
                          Exon5
Monkey      CCCGATTACAATACTTGAGCATCTGTAACACGGGCATCAGAAAGTTTCCAGATGTTACGA
Sequenced   ---------------------------------------------TTCC----GTTTCGG
Human       CCCGATTAAAATACTTGAGCATCTGTAACACAGGCATCAGAAAGTTTCCAGATGTTACGA
                                                         ****    *** ** 

                                           Exon6
Monkey      AGATCTTGTCCTCTGAATTAAATTTCATTCTGGAAATTTGTGATAACTTACACATAACCA
Sequenced   AGATCTTGTCCTCTGAATTAAATTTCATTCTGGAAATTTGTGATAACTTACACATAACCC
Human       AGGTCTTCTCCTCTGAATCAAATTTCATTCTGGAAATTTGTGATAACTTACACATAACCA
            ** **** ********** **************************************** 

                                                             Exon7
Monkey      CCATACCAGGAAATGCTTTTCAAGGGATGAATAATGAATCTGTAACACTCAAACTATATG
Sequenced   CCATAGCAGGAAATGCTTTTCAAGGGATGAATAATGAATCTGTAACACTCAAACTATATG
Human       CCATACCAGGAAATGCTTTTCAAGGGATGAATAATGAATCTGTAACACTCAAACTATATG
            ***** ******************************************************

Monkey      GAAATGGATTTGAAGAAGTACAAAGTCATGCGTTCAATGGGACGACACTGATTTCACTGG
Sequenced   GAAATGGATTTGAAGAAGTACAAAGTCATGCGGTCAATGGGACGACCCTGATTTCACTGG
Human       GAAATGGATTTGAAGAAGTACAAAGTCATGCATTCAATGGGACGACACTGACTTCACTGG
            *******************************  ************* **** ********
            Exon8
Monkey      AGCTAAAGGAAAACATACATCTGGAAAAGATGCACAATGGAGCCTTCCGTGGGGCCACGG
Sequenced   AGCTAAAGGAAAACATACATCTGGAAAAGATGCACAATGGAGCCTTCCGTGGGGCCACGG
Human       AGCTAAAGGAAAACGTACATCTGGAGAAGATGCACAATGGAGCCTTCCGTGGGGCCACAG
            ************** ********** ******************************** *
                         Exon9
Monkey      GGCCGAAAATCTTGGATATTTCTTCCACCAAATTGCAGGCCCTGCCGAGCTATGGCCTAG
Sequenced   GGCCGAAAATCTTGGATATTTCTTCCACCAAATTGCAGGCCCTGCCGAGCTATGGCCTAG
Human       GGCCGAAAACCTTGGATATTTCTTCCACCAAATTGCAGGCCCTGCCGAGCTATGGCCTAG
            ********* **************************************************

Monkey      AGTCCATTCAGACGCTAATTGCCACGTCATCCTATTCTCTAAAAAAATTGCCATCGAGAG
Sequenced   AGTCCATTCAGACGCTAATTGCCACGTCATCCTATTCTCTAAAAAAATTGCCATCGAGAG
Human       AGTCCATTCAGAGGCTAATTGCCACGTCATCCTATTCTCTAAAAAAATTGCCATCAAGAG
            ************ ****************************************** ****

Monkey      AAAAATTTGCCAATCTCCTGGAAGCCACGTTGACTTACCCCAGCCACTGCTGTGCTTTTA
Sequenced   AAAAATTTGCCAATCTCCTGGAAGCCACGTTGACTTACCCCAGCCACTGCTGTGCTTTTA
Human       AAACATTTGTCAATCTCCTGGAGGCCACGTTGACTTACCCCAGCCACTGCTGTGCTTTTA
            *** ***** ************ *************************************
                               Exon10
Monkey      GAAACTTGCCAACAAAAGAACAGAATTTTTCACTTTCCATTTCTGAAAACTTTTCCAAAC
Sequenced   GAAACTTGCCAACAAAAGAACAGAATTTTTCACTTTCCATTTCTGAAAACTTTTCCAAAC
Human       GAAACTTGCCAACAAAAGAACAGAATTTTTCACATTCCATTTCTGAAAACTTTTCCAAAC
            ********************************* **************************
                                                  Exon11
Monkey      AATGTGAAAGCACAGTAAGGAAACTGAATAATAAAACATTTTATTCTGCCATGCTTGCTG
Sequenced   AATGTGAAAGCACAGTAAGGAAACTGAATAATAAAACATTTTATTCTGCCATGCTTGCTG
Human       AATGTGAAAGCACAGTAAGGAAAGTGAATAACAAAACACTTTATTCTTCCATGCTTGCTG
            *********************** ******* ****** ******** ************

Monkey      AGAGTGAACTGAGTGGCTGGGACTATGAATATGGTTTCTGCTTACCCAAGACACCCCGAT
Sequenced   AGAGTGAACTGAGTGGCTGGGACTATGAATATGGTTTCTGCTTACCCAAGACACCCCGAT
Human       AGAGTGAACTGAGTGGCTGGGACTATGAATATGGTTTCTGCTTACCCAAGACACCCCGAT
            ************************************************************

Monkey      GTGCTCCTGAACCAGATGCTTTTAATCCCTGTGAAGATATTATGGGCTATGACTTCCTTA
Sequenced   GTGCTCCTGAACCAGATGCTTTTAATCCCTGTGAAGATATTATGGGCTATGACTTCCTTA
Human       GTGCTCCTGAACCAGATGCTTTTAATCCCTGTGAAGATATTATGGGCTATGACTTCCTTA
            ************************************************************

Monkey      GGGTCCTGATTTGGCTGATTAATATTCTAGCCATCATGGGAAACATGACTGTTCTTTTTG
Sequenced   GGGTCCTGATTTGGCTG-------------------------------------------
Human       GGGTCCTGATTTGGCTGATTAATATTCTAGCCATCATGGGAAACATGACTGTTCTTTTTG
            *****************
```

Figure S2: (B) Sequencing results for LH/CGR lower band (532 bp) obtained using multiple primer pair F2-R2 (exon 5-11)

(B) LH/CGR Lower band

```
                             Exon5
Monkey      CCCGATTACAATACTTGAGCATCTGTAACACGGGCATCAGAAAGTTTCCAGATGTTACGA
Sequenced   ----------------------------------------------------NGAGTCGA
Human       CCCGATTAAAATACTTGAGCATCTGTAACACAGGCATCAGAAAGTTTCCAGATGTTACGA
                                                             *   ***

                                           Exon6
Monkey      AGATCTTGTCCTCTGAATTAAATTTCATTCTGGAAATTTGTGATAACTTACACATAACCA
Sequenced   GA--CTTGTCCTCTGAATTAA-TTTCATTCTGGAAATTTGTGATAACTTACACATAACCA
Human       AGGTCTTCTCCTCTGAATCAAATTTCATTCTGGAAATTTGTGATAACTTACACATAACCA
                *** ********** ** **************************************

                                                             Exon7
Monkey      CCATACCAGGAAATGCTTTTCAAGGGATGAATAATGAATCTGTAACACTCAAACTATATG
Sequenced   CCATACCAGGAAATGCTTTTCAAGGGATGAATAATGAATCTGTAACACTCAAACTATATG
Human       CCATACCAGGAAATGCTTTTCAAGGGATGAATAATGAATCTGTAACACTCAAACTATATG
            ************************************************************

Monkey      GAAATGGATTTGAAGAAGTACAAAGTCATGCGTTCAATGGGACGACACTGATTTCACTGG
Sequenced   GAAATGGATTTGAAGAAGTACAAAGTCATGCGTTCAATGGGACGACACTGATTTCACTGG
Human       GAAATGGATTTGAAGAAGTACAAAGTCATGCATTCAATGGGACGACACTGACTTCACTGG
            ******************************* ******************* ********
            Exon8
Monkey      AGCTAAAGGAAAACATACATCTGGAAAAGATGCACAATGGAGCCTTCCGTGGGGCCACGG
Sequenced   AGCTAAAGGAAAACATACATCTGGAGAAGATGCACAATGGAGCCTTCCGTGGGGCCACGG
Human       AGCTAAAGGAAAACGTACATCTGGAGAAGATGCACAATGGAGCCTTCCGTGGGGCCACAG
            ************** ********** ******************************** *
                         Exon9
Monkey      GGCCGAAAATCTTGGATATTTCTTCCACCAAATTGCAGGCCCTGCCGAGCTATGGCCTAG
Sequenced   GGCCGAAAATCTT-----------------------------------------------
Human       GGCCGAAAACCTTGGATATTTCTTCCACCAAATTGCAGGCCCTGCCGAGCTATGGCCTAG
            ********* ***

Monkey      AGTCCATTCAGACGCTAATTGCCACGTCATCCTATTCTCTAAAAAAATTGCCATCGAGAG
Sequenced   ------------------------------------------------------------
Human       AGTCCATTCAGAGGCTAATTGCCACGTCATCCTATTCTCTAAAAAAATTGCCATCAAGAG

Monkey      AAAAATTTGCCAATCTCCTGGAAGCCACGTTGACTTACCCCAGCCACTGCTGTGCTTTTA
Sequenced   ------------------------------------------------------------
Human       AAACATTTGTCAATCTCCTGGAGGCCACGTTGACTTACCCCAGCCACTGCTGTGCTTTTA

                               Exon10
Monkey      GAAACTTGCCAACAAAAGAACAGAATTTTTCACTTTCCATTTCTGAAAACTTTTCCAAAC
Sequenced   -------------------ACAGAATTTTTCACTTTCCATTTCTGAAAACTTTTCCAAAC
Human       GAAACTTGCCAACAAAAGAACAGAATTTTTCACATTCCATTTCTGAAAACTTTTCCAAAC
                               ************** **************************
                                                  Exon11
Monkey      AATGTGAAAGCACAGTAAGGAAACTGAATAATAAAACATTTTATTCTGCCATGCTTGCTG
Sequenced   AATGTGAAAGCACAGTAAGGAAACTGAATAATAAAACATTTTATTCTGCCATGCTTGCTG
Human       AATGTGAAAGCACAGTAAGGAAAGTGAATAACAAAACACTTTATTCTTCCATGCTTGCTG
            *********************** ******* ****** ******** ************

Monkey      AGAGTGAACTGAGTGGCTGGGACTATGAATATGGTTTCTGCTTACCCAAGACACCCCGAT
Sequenced   AGAGTGAACTGAGTGGCTGGGACTATGAATATGGTTTCTGCTTACCCAAGACACCCNGAT
Human       AGAGTGAACTGAGTGGCTGGGACTATGAATATGGTTTCTGCTTACCCAAGACACCCCGAT
            ******************************************************** ***

Monkey      GTGCTCCTGAACCAGATGCTTTTAATCCCTGTGAAGATATTATGGGCTATGACTTCCTTA
Sequenced   GTGCTCCTGAACCAGATGCTTTTAATCCCTGTGAAGATATTATGGGCTATGACTTCCTTA
Human       GTGCTCCTGAACCAGATGCTTTTAATCCCTGTGAAGATATTATGGGCTATGACTTCCTTA
            ************************************************************

Monkey      GGGTCCTGATTTGGCTGATTAATATTCTAGCCATCATGGGAAACATGACTGTTCTTTTTG
Sequenced   GGGTCCTGATTTGGCTGA------------------------------------------
Human       GGGTCCTGATTTGGCTGATTAATATTCTAGCCATCATGGGAAACATGACTGTTCTTTTTG
            ******************
```
