# Supplementary figures and images for: Involvement of Src family of kinases and cAMP phosphodiesterase in the luteinizing hormone/chorionic gonadotropin receptor-mediated signaling in the corpus luteum of monkey
Source: Reprod Biol Endocrinol. 2012 Mar 29;10:25. doi: 10.1186/1477-7827-10-25 (PMC3353251; doi:10.1186/1477-7827-10-25)

**Figure S3: Characterization of FSHR mRNA in the monkey CL**

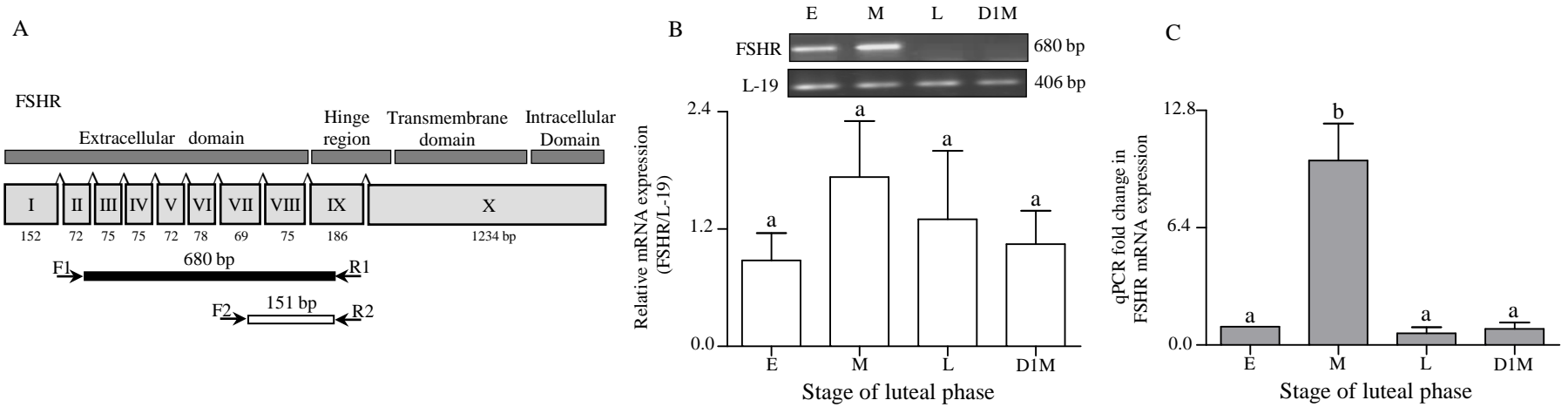

Supplement: Additional file 5 — Figure S3: (A) Schematic representation of FSHR gene depicting exons, various structural domains formed by exons and number of nucleotides in each exon region. The arrows indicate the primer set representing position of forward primers (F1&F2) and reverse primers (R1&R2) designed around the extracellular domain and hinge regions to detect FSHR mRNA transcripts (sizes 680 and 151 bp). (B) Semi-quantitative RT-PCR analysis to determine FSHR mRNA expression in the monkey CL during different stages of the luteal phase (E: early, M: mid, L: late and D1M: day 1 of menses). L-19 mRNA was used as internal control and the relative expression was calculated following densitometric analysis. Each bar represents mean ± SEM values (n = 3 CL/stage). No significant differences (p > 0.05; denoted by the same letter on individual bars) in expression was seen throughout the luteal phase. (C) The qPCR analysis for FSHR mRNA expression in the monkey CL during different stages of the luteal phase. The fold change in mRNA expression at each stage CL compared to early stage is represented in each bar as mean ± SEM values (n = 3 CL/stage). FSHR expression was high at mid luteal phase, but the expressions at late and D1M were low. Individual bars with different letters are significantly different (p < 0.05). [file 1477-7827-10-25-S5.PDF]

**Figure S4: Expression of PDE4D mRNA isoforms in the monkey CL**

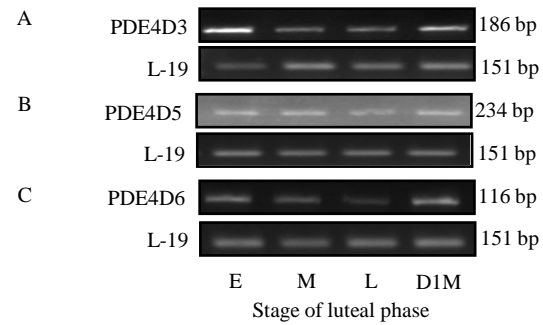

Supplement: Additional file 6 — Figure S4: Semi-quantitative RT-PCR analysis of PDE4D isoforms [PDE4D3 (A), PDE4D5 (B) and PDE4D6 (C)] was performed in CL tissue collected from monkeys during different stages of the luteal phase. The expression of housekeeping gene, L19, was used as the internal control. Shown here is a representative gel picture of PDE4D isoforms and L-19 PCR amplification products. [file 1477-7827-10-25-S6.PDF]

**Figure S5: Tissue cAMP levels in the monkey CL during different functional status**

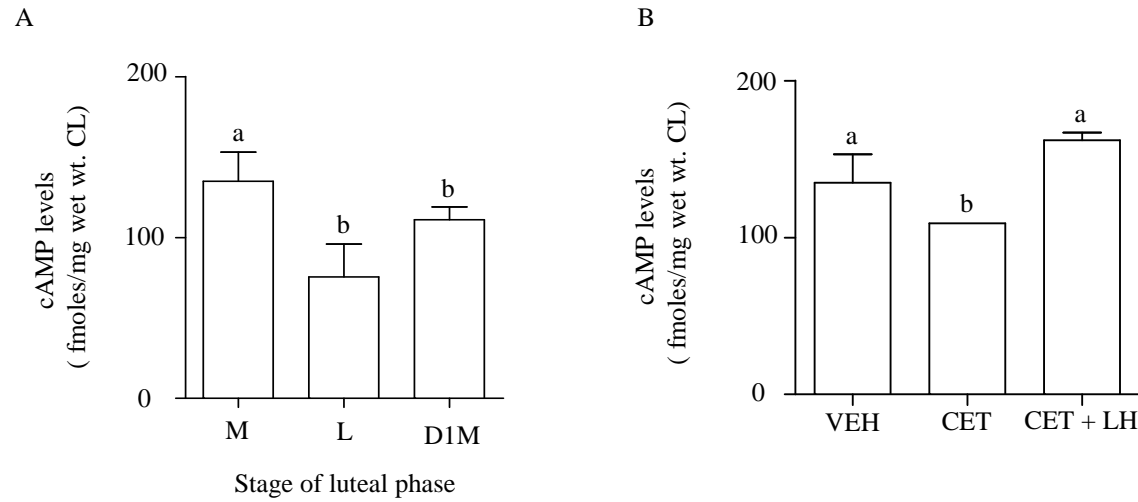

Supplement: Additional file 7 — Figure S5: (A) Tissue cAMP levels in the monkey CL collected during different stages of the luteal phase. Individual bars with different letters indicate statistical significance (p < 0.05). (B) Tissue cAMP levels from animals treated with Vehicle (VEH), CET and CET + LH (LH replacement). Individual bars with different letters indicate statistical significance (p < 0.05). [file 1477-7827-10-25-S7.PDF]
